# Hypermetabolic lymphadenopathy following the administration of COVID-19 vaccine and immunotherapy in a lung cancer patient: a case report

**DOI:** 10.1186/s13256-022-03660-9

**Published:** 2022-11-25

**Authors:** Shreya Tripathy, Nathaniel Alvarez, Shubham Jaiswal, Ryan Williams, Munaf Al-Khadimi, Sarah Hackman, William Phillips, Supreet Kaur, Sherri Cervantez, William Kelly, Josephine Taverna

**Affiliations:** 1 Department of Medicine, University of Texas Health Science Center, San Antonio, TX USA; 2 UT Health San Antonio, MD Anderson Mays Cancer Center, San Antonio, TX USA; 3 Department of Pathology and Laboratory Medicine, University of Texas Health Science Center, San Antonio, TX USA; 4 Department of Radiology, University of Texas Health Science Center, San Antonio, TX USA

**Keywords:** COVID-19, COVID-19 vaccine, Lung cancer, Lymphadenopathy, Case report

## Abstract

**Background:**

Given the current climate of the pandemic, lung cancer patients are especially vulnerable to complications from severe acute respiratory syndrome coronavirus 2 infection. As a high-risk population group, these patients are strongly advised to receive coronavirus disease 2019 vaccination in accordance with Center for Disease Control and Prevention guidelines to minimize morbidity and mortality. In recent years, immunotherapy has taken a preeminent role in the treatment of non-small cell lung cancer with dramatic improvement in overall survival. Reactive lymphadenopathy following the administration of a coronavirus disease 2019 vaccination can confound the radiographic interpretation of positron emission tomography–computed tomography or computed tomography scans from lung cancer patients receiving immunotherapy.

**Case presentation:**

Here, we present a case of a 61-year-old Caucasian female and former smoker who developed cervical, hilar, supraclavicular, mediastinal, and left retroauricular lymphadenopathy following her coronavirus disease 2019 booster vaccination. At the time, she had been receiving long-term immunotherapy for the treatment of advanced lung adenocarcinoma. Biopsy was pursued owing to concerns of treatment failure and confirmed recurrent malignancy.

**Conclusion:**

This case report highlights the importance of lymph node biopsies in lung cancer patients who present with contralateral lymphadenopathy following coronavirus disease 2019 vaccination to rule out tumor recurrence in this deserving patient population.

## Introduction

A new strain of coronavirus was first detected in Wuhan, China, during the winter of 2019. Now known as severe acute respiratory syndrome coronavirus 2 (SARS-CoV-2) or by its related disease nomenclature of coronavirus disease 2019 (COVID-19), this strain primarily infects the respiratory tract, causing a myriad of symptoms [1]. Clinical presentation can range from asymptomatic or mild symptoms (for example, flu-like illness, malaise, anosmia, ageusia) to severe symptomatology with acute respiratory distress syndrome, intravascular coagulopathy, and/or multiorgan failure resulting in death [1–4]. While the majority of infected patients (> 80%) only experience mild symptoms, some patients become hypoxic and require intensive-level care and mechanical ventilation to sustain oxygen requirements [5]. Populations particularly susceptible to COVID-19-related complications include lung cancer patients with active smoking history, chronic obstructive pulmonary disease, asthma, and/or interstitial lung disease [6, 7]. For these reasons, vaccinations are paramount for improving lung cancer survival outcomes during the pandemic [7]. In December 2020, the Food and Drug Administration (FDA) approved an emergency-use authorization for the mRNA-based Pfizer–BioNTech COVID-19 vaccine in individuals of more than 16 years of age [8]. Two additional vaccines, Moderna COVID-19 vaccine (mRNA-1273-based vaccine) and Janssen/Johnson & Johnson COVID-19 adenoviral vector vaccine (Ad26.COV2.S) were subsequently approved for emergency use by the FDA to reduce overall transmission risk [8]. Owing to the possibility of waning immunity and decreased efficacy against COVID-19 variants, the Center for Disease Control and Prevention (CDC) currently recommends vaccination with further booster series for lung cancer patients and other immunocompromised individuals [9]. Consequently, there have been reports of lymphadenopathy in active cancer patients following administration of the COVID-19 mRNA vaccine, which require close surveillance.

Within the last decade, immunotherapy has achieved unprecedented clinical efficacy in patients with non-small cell lung cancer. These immune checkpoint inhibitors are antibodies that specifically target cytotoxic T-lymphocyte-associated antigen 4 (CTLA-4) or programmed death 1 (PD-1) to block the signal that would have prevented activated T cells from attacking cancer cells, thus allowing the immune system to mount an antitumor immune response and clear the cancer [10–13]. In select cases, immunotherapy results in a delayed tumor shrinkage following an increase in tumor burden, making it challenging to discriminate pseudoprogression from true progression [14]. Further complicating matters, there has been limited information regarding the appearance of lymphadenopathy after COVID-19 vaccination as it relates to treatment response assessments in the setting of lung cancer patients on immunotherapy.

## Case description

A 61-year-old Caucasian female presented with an enlarging right-sided supraclavicular lymph node in 2017. Past medical history was notable for hypertension. Social history was notable for a 45-pack-year smoking history. Family history was noncontributory. She has no prior history of an autoimmune condition. A needle biopsy of the lymph node confirmed poorly differentiated carcinoma with PD-L1 expression of 20%. Immunohistochemistry stained weakly for progesterone receptor (estrogen receptor and HER-2 receptors were negative) raising initial concerns for breast cancer. However, no breast masses or axillary/intramammary lymph nodes had been detected on mammogram or computed tomography (CT) scans. An esophagogastroduodenoscopy revealed no malignancy. Initial positron emission tomography–computed tomography (PET–CT) scan was notable for fluorodeoxyglucose (FDG) avid lymph nodes in the mediastinum, bilateral pulmonary hilum, right infrahyoid jugular chain and a 3 cm tumor in the right upper lung, consistent with Stage IVA (T2N3M1a) lung carcinoma. She initiated chemotherapy with carboplatin and paclitaxel, but developed disease progression following three treatment cycles. At the time of progression, [^18^F] PET–CT revealed multiple intensely FDG avid bilateral pulmonary nodules and lymphadenopathy involving bilateral hilar, mediastinal, and supraclavicular regions encroaching upon the lower neck. She initiated second-line treatment with nivolumab (checkpoint inhibitor) 240 mg intravenous infusion every 2 weeks, achieving a partial response to therapy. Following 12 treatment cycles, there was a notable reduction in size of right upper lobe pulmonary nodule (1 cm) and decrease in FDG avidity of the confluent multi-station mediastinal, right hilar lymphadenopathy and bilateral supraclavicular lymph nodes. She continued immunotherapy for a total of 4 years with stable disease and without significant adverse events, except for grade 1 arthralgias affecting hands and feet. She tolerated immunotherapy well and had an excellent performance status (ECOG 0). During these 4 years, she underwent routine tumor surveillance with full physical exams, routine labs and imaging every 3–4 months, which revealed no changes in mediastinal and right hilar lymphadenopathy.

**Fig. 1:**
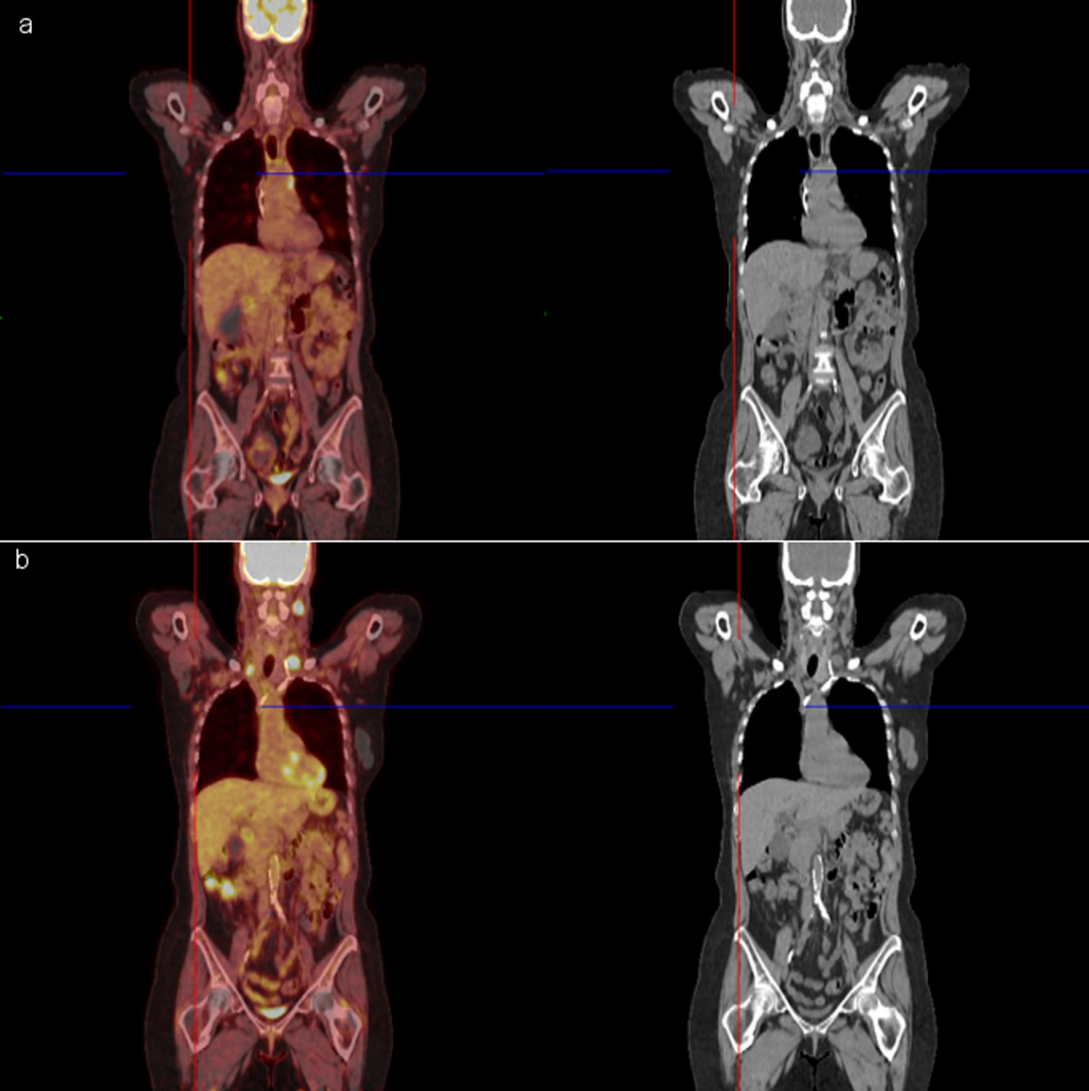
A 61-year-old woman with Stage IV carcinoma of the right lung receiving maintenance immunotherapy had PET–CT scan imaging (**a**) approximately 15 months prior to receiving her COVID-19 booster vaccination. PET–CT scan imaging of the same woman performed 2 weeks following COVID-19 booster vaccinations (**b**). Imaging reveals mixed response to treatment with a new left-sided level Xa retroauricular lymph node (1.5 × 1.8 cm, SUVmax 20), interval decrease in the size of the multilevel bilateral subcentimeter cervical chain lymph nodes without FDG uptake, and a right-sided level III lymph node measuring 1 cm with max SUV 8.0 (previously 8 mm and SUVmax 8.5). Interval increase in the size of the hypermetabolic level IVa lymph node measuring 1.8 cm with SUVmax 14.8 (previously 8 mm, SUV 5.3); and new left supraclavicular hypermetabolic lymph node measuring 8 mm with SUVmax 14.6

At the recommendation of her oncologist and following CDC guidelines, the patient went on to receive the Pfizer–BioNTech COVID-19 vaccination two-shot series in the right deltoid muscle in March 2021 and had experienced no symptoms except for left-sided retroauricular lymphadenopathy. A CT chest performed 3 months prior to her COVID-19 vaccine revealed interval decrease in the right lower lobe nodular opacity, unchanged 10 mm nodular opacity in the left lower lobe, and unchanged mediastinal and right hilar lymphadenopathy, with the largest right paratracheal lymph node measuring 1.5 cm. Two weeks following her vaccination, she developed a palpable lymph node behind her left ear. Follow-up [^18^F] PET–CT scan revealed new left supraclavicular hypermetabolic lymph node [8 mm with maximum standardized uptake value (SUVmax) 14.8], a new left-sided retroauricular lymph node with intense FDG activity (1.8 cm with SUVmax 20) (Fig. 1) and decrease in size and FDG activity of cervical lymph nodes. The FDG avid lymph nodes raised concern for potential treatment failure.

**Fig. 2:**
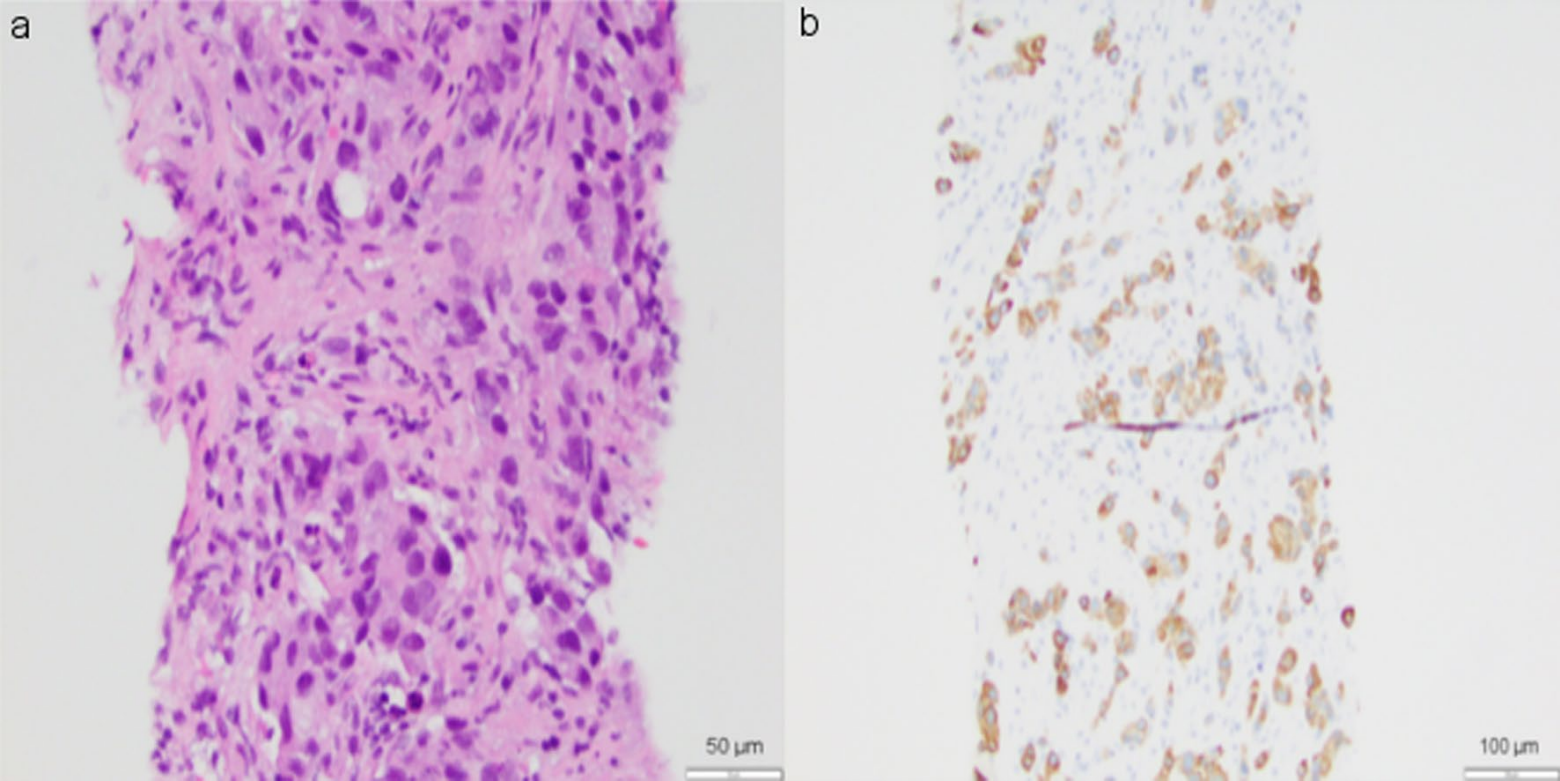
Preauricular lymph node tissue obtained by ultrasound-guided core needle biopsy. Hematoxylin and eosin staining (200×) reveals poorly differentiated carcinoma (**a**). Immunohistochemical studies show malignant cells are diffusely and strongly positive for cytokeratin 7 (**b**)

At the time of her follow-up appointment with her medical oncologist, she noticed a significant reduction in the size of her left retroauricular lymph node. Physical exam was notable for palpable and non-tender left supraclavicular lymph node and left-sided retroauricular lymph node. Labs revealed a normal complete blood count (CBC, white blood cell count 7300 per microliter, hemoglobin 14.2 g/dL, platelet count 218,000 per microliter), normal renal and liver panels, and normal thyroid function tests. Although subjectively the adenopathy was improving, she underwent an ultrasound-guided core needle biopsy of the left preauricular lymph node, which confirmed tumor recurrence with poorly differentiated carcinoma. Immunohistochemistry (IHC) revealed that malignant cells were diffusely and strongly positive for cytokeratin 7, but weakly positive for GATA3 and negative for CK20, TTF-1, Napsin A, CDX2, PAX8, uroplakin, mammoglobin, estrogen receptor (ER), progesterone receptor (PR), GCDFP15, and p63 (Fig. 2). CancerTypeID molecular profiling determined with 90% probability that tumor of origin was lung. Following discussions regarding her diagnosis, prognosis, and systemic treatment options, patient agreed to pursue chemotherapy to delay and/or prevent tumor growth and spread.

She initiated second-line chemotherapy with docetaxel 75 mg/m^2^ intravenous infusion every 3 weeks and tolerated treatment well, except for grade 1 fatigue. Following three treatment cycles, she had achieved a partial treatment response and her CT scans revealed interval decrease in size of multiple supraclavicular, mediastinal, and R hilar lymphadenopathy. She transitioned to a phase 2 study of oradoxel, an oral dosage form of docetaxel with a novel P-glycoprotein inhibitor. She continued to respond well to treatment after three cycles and continued on study without treatment delays.

## Discussion

Many factors confound the interpretation of radiographic imaging for cancer patients receiving systemic therapy during the COVID-19 pandemic. Like other strongly immunogenic vaccines, COVID-19 vaccines can serve as strong antigenic stimuli resulting in unilateral adenopathy [15]. While clinical context remains a key determinant in the evaluation and management of patients with lung cancer, evolving information regarding COVID-19 vaccination has shaped the approach and level of caution. While our patient appeared to have a durable response to immunotherapy, new adenopathy after prolonged immunotherapy maintenance needed to be evaluated by histological exam to distinguish vaccine-associated hypermetabolic lymphadenopathy from local tumor recurrence. COVID-19 vaccination can contribute to regional lymphadenopathy, making it difficult to discern reactive lymphadenopathy from malignancy [16, 17]. In a SCOPUS review of 19 studies, 68 patients developed reactive lymphadenopathy within the first day and lasted 4–6 weeks following their first or second COVID-19 vaccination [18]. As observed in our patient, the Pfizer–BioNTech vaccine resulted in the most cases of lymphadenopathy (44.1%) compared with Moderna (25%) and Oxford-AstraZeneca (1.5%) COVID-19 vaccines [18]. Most common sites of lymphadenopathy involved ipsilateral axillary, supraclavicular, cervical, and subpectoral nodal regions. More than 80% of these patients had a prior or active cancer history with most cases presenting as breast cancer (*n* = 40 cases). In this study, only four cases of lung cancer were found [18]. In another study, hypermetabolic lymphadenopathy was detected by PET scan in 266 cases out of 728 cancer patients following their COVID-19 vaccination. From these cases, 17 cancer patients were found to have malignancy-associated hypermetabolic lymphadenopathy and 49 patients had equivocal results. Approximately 50% of patients had not developed hypermetabolic lymphadenopathy following their COVID-19 vaccination [19]. These studies highlight the importance of vaccinating patients in the arm contralateral to the tumor’s expected nodal drainage when feasible. For example, in a patient with a localized tumor in the left lung, the COVID-19 vaccine should be administered in the right arm to minimize confusion and assist in the radiologic interpretation of PET scans. Our patient developed unilateral adenopathy, which is typical for vaccine associated lymphadenopathy, but the distribution of lymphadenopathy forewarned lung cancer progression and prompted a diagnostic biopsy. COVID-19-vaccine-associated hypermetabolic lymphadenopathy may last up to 10 weeks [21]. For these reasons, biopsy of enlarging and hypermetabolic lymph nodes is warranted to rule out tumor progression. To conclude, physicians should consider delaying PET scan imaging for at least 10 weeks after vaccination in lung cancer patients to allow resolution of lymphadenopathy [20, 21]. If a patient has cancer with laterality, the vaccine should be administered in the contralateral arm to avoid confounding radiologic interpretation [22, 23]. Alternatively, injecting COVID-19 vaccine in thigh muscle instead of the deltoid muscle of lung cancer patients might be a reasonable option.

Much remains to be discovered regarding the interplay between immunity from COVID-19 infection, COVID-19 vaccination, and the PD-1/PD-L1 axis responsible for cancer immune escape. Cancer patients being treated with checkpoint blockade (or PD-L1 axis blockade) can develop decreased SARS-CoV-2 antibody titers after vaccination resulting in the CDC recommendation for a third booster in this population [24]. Furthermore, studies have shown that several co-inhibitory molecules (PD-L1, CTLA-4, TIM-3, LAG3, VISTA, TIGIT) are upregulated on T cells in patient with COVID-19 infections, and this might negatively interfere with antitumor immune responses induced by checkpoint inhibitors [25, 26]. One study has suggested that while PD-1 expressing CD8^+^ T cells from COVID-19 patients are still functional, these T cells become exhausted [27, 28]. There are currently several ongoing clinical trials examining the efficacy of PD-L1 axis blockade to combat deleterious effects of COVID-19-induced T-cell exhaustion [29]. Whether the immune response generated by COVID-19 vaccination produces T-cell exhaustion in cancer patients (as seen with chronic infections) to sufficiently dampen immune checkpoint blockade in patients being treated for malignancy remains unknown. A multicenter study of 2084 Chinese patients treated with immune checkpoint inhibitors showed no difference in overall response rate between vaccinated and unvaccinated patients, although immune-related adverse events were more frequently observed in vaccinated patients [30]. Ultimately, clinical context in light of our evolving understanding of COVID-19 remains the driving factor for evaluation and management of oncologic patients.

## Conclusions

Lung cancer patients receiving immunotherapy require a higher level of tumor surveillance during the COVID-19 pandemic because tumor progression can masquerade as vaccine-associated hypermetabolic lymphadenopathy. Whenever possible, lung cancer patients should be advised to get their COVID-19 vaccine in the arm contralateral to the tumor (or deltoid muscle), which may aid in the radiographic assessment of these patients.

## Data Availability

Not applicable.

## References

[1] Umakanthan S, Sahu P, Ranade AV, Bukelo MM, Rao JS, Abrahao-Machado LF (2020). Origin, transmission, diagnosis and management of coronavirus disease 2019 (COVID-19). Postgrad Med J.

[2] Zhou F, Yu T, Du R, Fan G, Liu Y, Liu Z (2020). Clinical course and risk factors for mortality of adult inpatients with COVID-19 in Wuhan, China: a retrospective cohort study. Lancet.

[3] Faggiano P, Bonelli A, Paris S, Milesi G, Bisegna S, Bernardi N (2020). Acute pulmonary embolism in COVID-19 disease: preliminary report on seven patients. Int J Cardiol.

[4] Zhang L, Zhu F, Xie L, Wang C, Wang J, Chen R (2020). Clinical characteristics of COVID-19-infected cancer patients: a retrospective case study in three hospitals within Wuhan, China. Ann Oncol.

[5] Kommoss FKF, Schwab C, Tavernar L, Schreck J, Wagner WL, Merle U (2020). The pathology of severe COVID-19-related lung damage. Dtsch Arztebl Int.

[6] Passaro A, Bestvina C, Velez Velez M, Garassino MC, Garon E, Peters S (2021). Severity of COVID-19 in patients with lung cancer: evidence and challenges. J Immunother Cancer.

[7] Dai M, Liu D, Liu M, Zhou F, Li G, Chen Z (2020). Patients with cancer appear more vulnerable to SARS-CoV-2: a multicenter study during the COVID-19 outbreak. Cancer Discov.

[8] Food and Drug Administration US. COVID-19 vaccinations 2022. https://www.fda.gov/emergency-preparedness-and-response/coronavirus-disease-2019-covid-19/covid-19-vaccines. Updated 7 Jan 2022; Accessed 1 Jan 2022.

[9] Centers for Disease Control and Prevention US. COVID-19 vaccines for moderately or severely immunocompromised people 2022. https://www.cdc.gov/coronavirus/2019-ncov/vaccines/recommendations/immuno.html. Updated 7 Jan 2022; Accessed 9 Jan 2022.

[10] Gandhi L, Rodríguez-Abreu D, Gadgeel S, Esteban E, Felip E, De Angelis F (2018). Pembrolizumab plus chemotherapy in metastatic non-small-cell lung cancer. N Engl J Med.

[11] Rodríguez-Abreu D, Powell SF, Hochmair MJ, Gadgeel S, Esteban E, Felip E (2021). Pemetrexed plus platinum with or without pembrolizumab in patients with previously untreated metastatic nonsquamous NSCLC: protocol-specified final analysis from KEYNOTE-189. Ann Oncol.

[12] Herbst RS, Baas P, Kim DW, Felip E, Pérez-Gracia JL, Han JY (2016). Pembrolizumab versus docetaxel for previously treated, PD-L1-positive, advanced non-small-cell lung cancer (KEYNOTE-010): a randomised controlled trial. Lancet.

[13] Paz-Ares L, Luft A, Vicente D, Tafreshi A, Gümüş M, Mazières J (2018). Pembrolizumab plus chemotherapy for squamous non-small-cell lung cancer. N Engl J Med.

[14] Frelaut M, du Rusquec P, de Moura A, Le Tourneau C, Borcoman E (2020). Pseudoprogression and hyperprogression as new forms of response to immunotherapy. BioDrugs.

[15] Cocco G, Delli Pizzi A, Taraschi AL, Boccatonda A, Corvino A, Ucciferri C (2022). Atypical sites of lymphadenopathy after anti-COVID-19 vaccine: ultrasound features. Medicina (Kaunas).

[16] Singh B, Kaur P, Kumar V, Maroules M (2021). COVID-19 vaccine induced axillary and pectoral lymphadenopathy on PET scan. Radiol Case Rep.

[17] Doss M, Nakhoda SK, Li Y, Yu JQ (2021). COVID-19 vaccine-related local FDG uptake. Clin Nucl Med.

[18] Keshavarz P, Yazdanpanah F, Rafiee F, Mizandari M (2021). Lymphadenopathy following COVID-19 vaccination: imaging findings review. Acad Radiol.

[19] Cohen D, Krauthammer SH, Wolf I, Even-Sapir E (2021). Hypermetabolic lymphadenopathy following administration of BNT162b2 mRNA Covid-19 vaccine: incidence assessed by [(18)F]FDG PET-CT and relevance to study interpretation. Eur J Nucl Med Mol Imaging.

[20] McIntosh LJ, Bankier AA, Vijayaraghavan GR, Licho R, Rosen MP (2021). COVID-19 vaccination-related uptake on FDG PET/CT: an emerging dilemma and suggestions for management. Am J Roentgenol.

[21] McIntosh LJ, Rosen MP, Mittal K, Whalen GF, Bathini VG, Ali T (2021). Coordination and optimization of FDG PET/CT and COVID-19 vaccination; lessons learned in the early stages of mass vaccination. Cancer Treat Rev.

[22] Lehman CD, Lamb LR, D'Alessandro HA (2021). Mitigating the impact of coronavirus disease (COVID-19) vaccinations on patients undergoing breast imaging examinations: a pragmatic approach. Am J Roentgenol.

[23] Becker AS, Perez-Johnston R, Chikarmane SA, Chen MM, El Homsi M, Feigin KN (2021). Multidisciplinary recommendations regarding post-vaccine adenopathy and radiologic imaging: radiology scientific expert panel. Radiology.

[24] Lasagna A, Lilleri D, Agustoni F, Percivalle E, Borgetto S, Alessio N (2022). Analysis of the humoral and cellular immune response after a full course of BNT162b2 anti-SARS-CoV-2 vaccine in cancer patients treated with PD-1/PD-L1 inhibitors with or without chemotherapy: an update after 6 months of follow-up. ESMO Open.

[25] Khalifehzadeh-Esfahani Z, Fattahi S, Heidari Haratemeh Z, Jafarinia M (2022). The role of immune regulatory molecules in COVID-19. Viral Immunol.

[26] Al-Mterin MA, Alsalman A, Elkord E (2022). Inhibitory immune checkpoint receptors and ligands as prognostic biomarkers in COVID-19 patients. Front Immunol.

[27] Rha MS, Jeong HW, Ko JH, Choi SJ, Seo IH, Lee JS (2021). PD-1-expressing SARS-CoV-2-specific CD8(+) T cells are not exhausted, but functional in patients with COVID-19. Immunity.

[28] Mortezaee K, Majidpoor J (2022). CD8(+) T cells in SARS-CoV-2 induced disease and cancer-clinical perspectives. Front Immunol.

[29] Vivarelli S, Falzone L, Leonardi GC, Salmeri M, Libra M (2021). Novel insights on gut microbiota manipulation and immune checkpoint inhibition in cancer (Review). Int J Oncol.

[30] Mei Q, Hu G, Yang Y, Liu B, Yin J, Li M (2022). Impact of COVID-19 vaccination on the use of PD-1 inhibitor in treating patients with cancer: a real-world study. J Immunother Cancer.

